# Unbiased proteomics and multivariable regularized regression techniques identify SMOC1, NOG, APCS, and NTN1 in an Alzheimer’s disease brain proteomic signature

**DOI:** 10.1038/s41514-023-00112-6

**Published:** 2023-07-06

**Authors:** Jackson A. Roberts, Vijay R. Varma, Julián Candia, Toshiko Tanaka, Luigi Ferrucci, David A. Bennett, Madhav Thambisetty

**Affiliations:** 1 Clinical and Translational Neuroscience Section, Laboratory of Behavioral Neuroscience, National Institute on Aging, National Institutes of Health, Baltimore, MD 21224 USA; 2 Columbia University Vagelos College of Physicians and Surgeons, New York, NY 10032 USA; 3 Longitudinal Studies Section, Translational Gerontology Branch, National Institute on Aging, National Institutes of Health, Baltimore, MD 21224 USA; 4 Rush Alzheimer’s Disease Center, Rush University Medical Center, Chicago, IL 60612 USA

**Keywords:** Alzheimer's disease, Cognitive ageing

## Abstract

Advancements in omics methodologies have generated a wealth of high-dimensional Alzheimer’s disease (AD) datasets, creating significant opportunities and challenges for data interpretation. In this study, we utilized multivariable regularized regression techniques to identify a reduced set of proteins that could discriminate between AD and cognitively normal (CN) brain samples. Utilizing *eNetXplorer*, an R package that tests the accuracy and significance of a family of elastic net generalized linear models, we identified 4 proteins (SMOC1, NOG, APCS, NTN1) that accurately discriminated between AD (*n* = 31) and CN (*n* = 22) middle frontal gyrus (MFG) tissue samples from Religious Orders Study participants with 83 percent accuracy. We then validated this signature in MFG samples from Baltimore Longitudinal Study of Aging participants using leave-one-out logistic regression cross-validation, finding that the signature again accurately discriminated AD (*n* = 31) and CN (*n* = 19) participants with a receiver operating characteristic curve area under the curve of 0.863. These proteins were strongly correlated with the burden of neurofibrillary tangle and amyloid pathology in both study cohorts. We additionally tested whether these proteins differed between AD and CN inferior temporal gyrus (ITG) samples and blood serum samples at the time of AD diagnosis in ROS and BLSA, finding that the proteins differed between AD and CN ITG samples but not in blood serum samples. The identified proteins may provide mechanistic insights into the pathophysiology of AD, and the methods utilized in this study may serve as the basis for further work with additional high-dimensional datasets in AD.

## Introduction

Alzheimer’s disease (AD) remains the most common neurodegenerative disorder and cause of dementia among the elderly, yet causal mechanisms driving sporadic AD remain elusive. Previously, much AD research has focused on hypothesis-driven interrogation of molecular mechanisms, which contributed to the identification of the central roles of amyloid and tau in disease, as well as the genetic association of the *APOE ε4* allele with sporadic AD. However, targeted and hypothesis-oriented research may preclude the discovery of novel molecular mechanisms underlying AD. The development of multiple unbiased omics methods and their application to the large-scale analyses of blood and brain tissue in AD holds promise for the identification of novel molecular mechanisms associated with AD pathogenesis.

Proteomics offers a particularly exciting opportunity for the study of disease mechanisms, as proteins represent the effector molecules of many upstream biological processes and additionally serve as the targets for the majority of known and potential pharmacological therapeutics. Additionally, advances in high throughput data acquisition have transformed proteomics into a field in which rich datasets are readily generated, resulting in an abundance of proteomic data for analysis^1^. As data become more abundant, the challenge for researchers is no longer quantification but rather analysis and interpretation of multi-dimensional datasets. In response, systems biology approaches have been elaborated, including network modeling analyses that seek to cluster variables using multiple methods to establish similarity distances between variables^2^. In proteomics, this may take the form of protein-protein interaction networks that can be generated for different disease states, including AD, which may be followed by pathway enrichment to identify classes of biological mechanisms altered in disease^3^. In AD, such approaches have contributed to a global understanding of processes associated with disease, proteostasis, immune response, cell signaling, mitochondrial function, metabolism, RNA binding and splicing, and several other biological pathways^4,5^.

While large-scale, co-expression-based approaches have made significant contributions to understanding altered biologic networks in disease states, studies focused on individual protein contributions to disease remain limited by methodologic heterogeneity^6^. Indeed, studies that examine individual protein differences in the context of disease are largely restricted to univariate analyses that do not take into account the complexity of data structures and inter-variable correlation that characterizes multi-dimensional datasets in small sample size studies. In this context, the elastic net^7^ offers a regularized generalized linear modeling and variable selection method that is flexible and accounts for multi-collinearity, allowing for the selection of a reduced set of features that are highly associated with the outcome. The eNetXplorer R package^8^ implemented functionality to sample different predictive elastic net models with a cross-validated approach that generates model significance and predictive scores. This framework has recently been applied to transcriptomic^9^, brain imaging^10^, and proteomic^11^ data, showing the ability to scan a high-dimensional feature space to find cross-validated signatures predictive of a disease state. Such signatures may, as a result, represent plausible drug targets or provide insight into disease pathomechanisms.

In this study, we undertook an unbiased proteomics approach in brain tissue samples to identify a set of proteins that could accurately discriminate between AD and cognitively normal (CN) individuals in two separate cohorts. We applied brain proteomic analyses in the inferior temporal gyrus (ITG) and middle frontal gyrus (MFG) in the Religious Orders Study (ROS) using regularized generalized elastic net modeling to develop an AD proteomic signature and then tested the discriminatory potential of this signature in the Baltimore Longitudinal Study of Aging (BLSA). We then compared the levels of proteins included in the signature in BLSA and ROS ITG and MFG brain tissue, as well as in blood serum samples collected at time of AD diagnosis, in order to determine if signature proteins are reflected in raw tissue level comparisons both within the signature brain region and additional tissue matrices. Lastly, we examined the association of blood serum and brain tissue levels of these proteins with the severity of AD pathology.

## Results

### Demographic characteristics

The demographic characteristics of participants who provided brain tissue samples are summarized in Table 1. In the BLSA sample, AD and CN groups did not differ significantly in age at death or sex. The AD group had a higher proportion of white participants (race) than the CN samples. As anticipated, AD and CN groups differed significantly in the severity of neuritic plaques (CERAD scores) and neurofibrillary tangles (Braak scores), with higher levels of pathology in the AD group.

**Table 1:** Cohort demographics.

Baltimore longitudinal study of aging (BLSA)			
	Total Sample *N* = 50	AD *N* = 31	CN *N* = 19
Age at death, mean (SD)	86.38 (10.70)†	88.46 (8.30)†	82.98 (13.31)
Age of onset, mean (SD)	-	78.81 (9.92)†	
Disease duration, mean (SD)	-	9.65 (4.47)†	
Sex, *n* (% female)	21 (42.00)†	15 (48.39)†	6 (31.58)
Race, *n* (% white)	48 (96.00)	31 (100)*	17 (89.47)*
CERAD, mean (SD)	1.78 (1.34)	2.77 (0.43)*	0.16 (0.38)*
Braak, mean (SD)	4.14 (1.69)	5.13 (1.12)*†	2.53 (1.12)*

Religious orders study (ROS)			
	Total Sample *N* = 53	AD *N* = 31	CN *N* = 22
Age at death, mean (SD)	90.58 (6.53)†	92.81 (5.45)*†	87.44 (6.75)*
Age of onset, mean (SD)	-	89.14 (5.71)†	-
Disease duration, mean (SD)	-	3.67 (2.94)†	-
Sex, *n* (% female)	40 (75.47)†	27 (87.10)*†	13 (59.09)*
Race, *n* (% white)	53 (100.00)	31 (100.00)	22 (100.00)
CERAD, mean (SD)	1.64 (1.26)	2.58 (0.50)*	0.32 (0.65)*
Braak, mean (SD)	3.89 (1.33)	4.65 (0.66)*†	2.82 (1.30)*

*p < 0.05 comparing AD to CN within a cohort.

^†^*p* < 0.05 comparing BLSA to ROS (i.e., AD in BLSA compared to AD in ROS).

In the ROS sample, all individuals included in the study were of white race. The AD group was older at the age of death and had a higher proportion of female (sex) participants compared to CN. As in BLSA, the AD group displayed higher CERAD and Braak scores, indicative of a higher burden of pathology in this group.

We also compared demographic characteristics across the BLSA and ROS cohorts. Considering the full sample, BLSA and ROS varied significantly in sex and age at death. Comparing BLSA AD participants to ROS AD participants, the BLSA AD group had an earlier age of disease onset, longer duration of disease, had a smaller proportion of female participants, and had higher Braak scores than the ROS AD group.

Additional details regarding the demographic characteristics of individuals with blood serum samples are provided in Supplementary Table 2.

### Protein signature evaluation in MFG

**Fig. 1:**
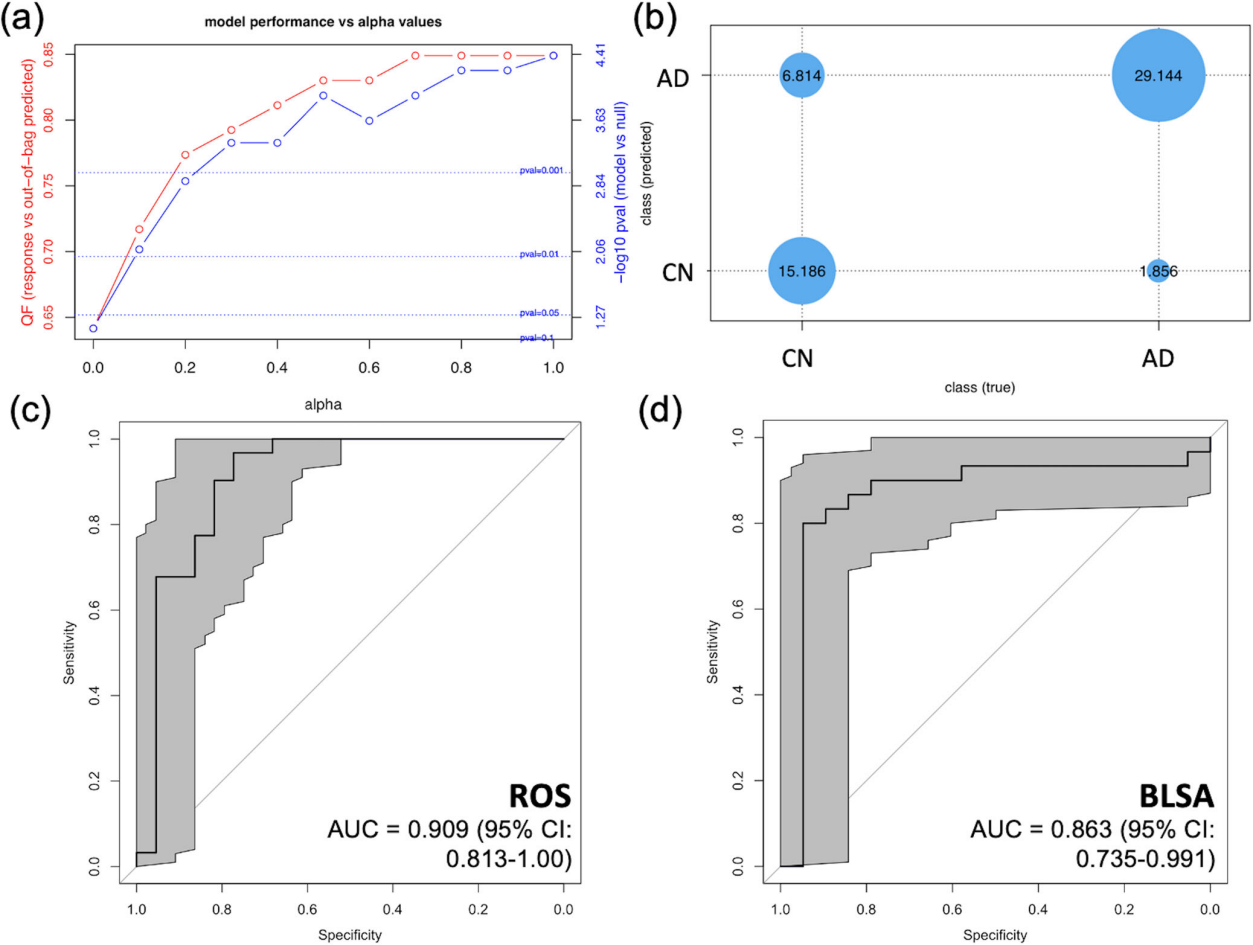
Establishment and evaluation of MFG proteomic signature. **a** Output of eNetXplorer model selection analyses, performed in ROS MFG. The x-axis indicates the model *alpha* value, a parameter that scales from the ridge (alpha = 0) to lasso (alpha = 1) regression. The red line indicates accuracy as quality function (QF), which represents the proportion of out-of-bag class (AD vs. CN) predictions that match the true class of each participant. The blue line indicates the model significance for each alpha value. **b** Contingency matrix comparing the out-of-bag predicted class and true class for the selected model. **c** Receiver operating characteristic (ROC) curve for leave-one-out cross-validation (LOOCV) of the 4 protein MFG signature in the ROS cohort, derived at the alpha = 1 level. Gray shading indicates the 95 percent confidence interval. **d** ROC curve for LOOCV of MFG samples from an independent cohort of BLSA participants. The area under the curve (AUC) is demonstrated with 95 percent confidence intervals. Gray shading indicates the 95 percent confidence interval.

We performed eNetXplorer analyses to develop a discriminatory protein signature in ROS MFG samples. In the MFG, the best-performing model (alpha = 1) discriminated between AD and CN brain samples with 83 percent accuracy (Fig. 1a, b). This model identified four proteins (secreted modular calcium-binding protein 1 (SMOC1), noggin (NOG), amyloid P component, serum (APCS), and netrin-1 (NTN1)) based upon the significance of feature frequency, among which SMOC1 displayed a positive feature coefficient—i.e., an increased level of this protein was associated with an increased probability of classification as AD (Supplementary Fig. 1a). Furthermore, these proteins displayed significant feature frequencies at all *alpha* levels tested, signifying stability of the signature across model stringencies (Supplementary Fig. 1d). These proteins were reassessed as a signature in a leave-one-out cross-validated logistic regression model, which, as expected, was able to accurately discriminate between ROS MFG AD and CN samples with AUC = 0.909 (95% CI: 0.813–1.00) (Fig. 1c). We then tested the ability of these proteins to discriminate between AD and CN MFG samples in the independent BLSA cohort. Remarkably, the protein signature accurately discriminated BLSA MFG samples, with AUC = 0.863 (95% CI: 0.735–0.991), as shown in Fig. 1d. As a result of this independent validation of the ROS MFG signature, these proteins were further evaluated in the remainder of the study.

We additionally tested the ability of the ROS-derived MFG signature to discriminate between AD and CN in BLSA ITG samples. The MFG signature failed to discriminate accurately between AD and CN participants in the BLSA ITG, with an AUC = 0.626 (95% CI: 0.428–0.825).

### Protein signature evaluation in ITG

**Fig. 2:**
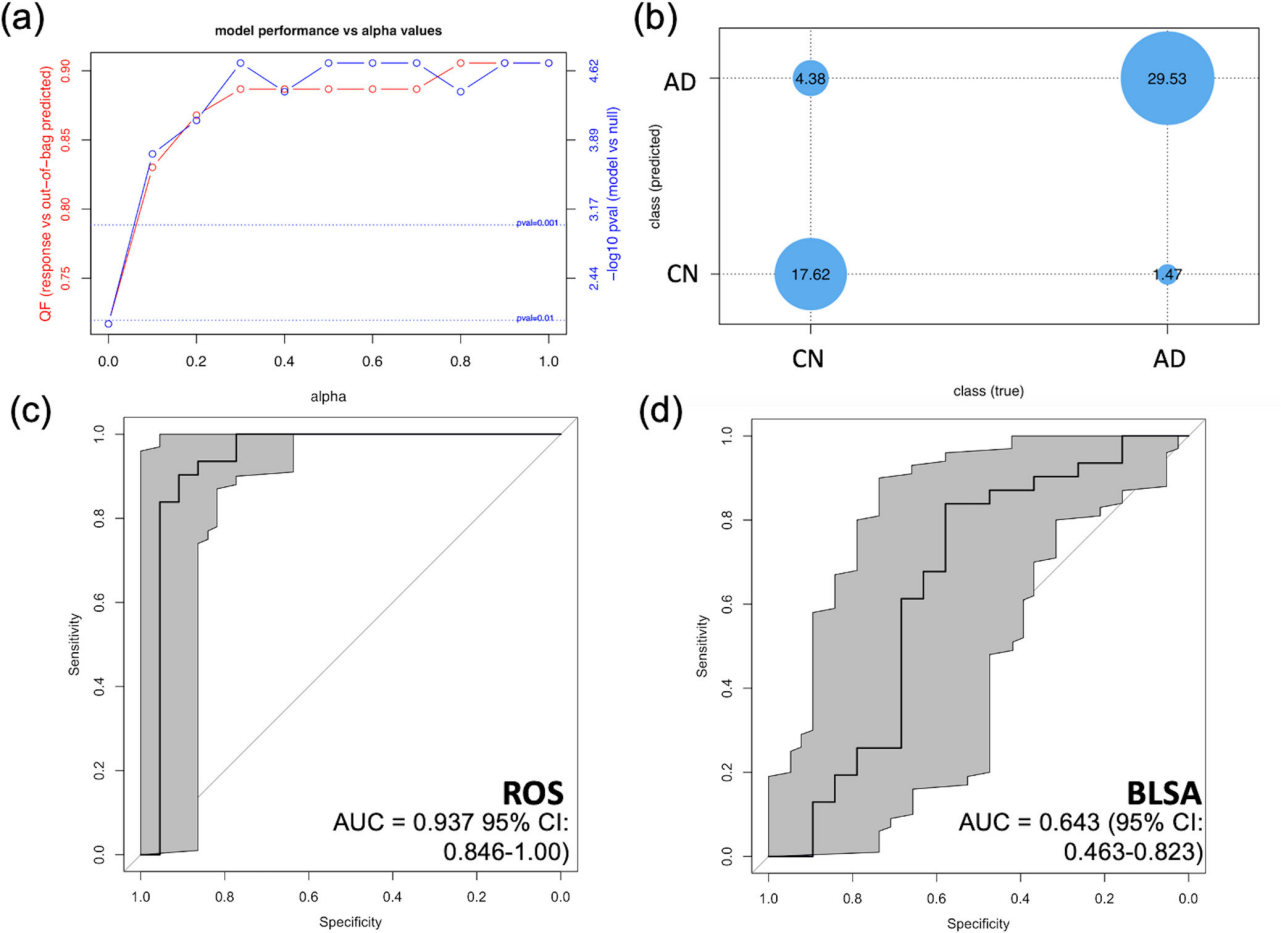
Establishment and evaluation of ITG proteomic signature. **a** Output of eNetXplorer model selection analyses, performed in ROS ITG. The x-axis indicates the model *alpha* value, a mixing parameter that scales continuously from the ridge (alpha = 0) to lasso (*alpha* = 1) regularized regression models. The red line indicates the out-of-bag prediction performance using accuracy as quality function (QF), which captures the proportion of class (AD vs. CN) predictions that match the true class of each participant. The blue line indicates the estimated model significance for each *alpha* value. **b** Contingency matrix comparing the out-of-bag predicted class and true class for the selected model (alpha = 1). **c** Receiver operating characteristic (ROC) curve for leave-one-out cross-validation (LOOCV) of the four protein ITG signature in the ROS cohort, derived at the *alpha* = 1 level. Gray shading indicates the 95 percent confidence interval. **d** ROC curve for LOOCV of ITG samples from an independent cohort of BLSA participants. The area under the curve (AUC) is demonstrated with 95 percent confidence intervals. Gray shading indicates the 95 percent confidence interval.

To establish an ITG proteomic signature, we performed eNetXplorer analyses in ROS ITG to determine which multivariable protein model best discriminates between AD and CN participants. In the ITG, the highest cross-validated model performance was obtained in the range alpha = 0.8–1, which discriminated between AD and CN ROS participants with 91 percent accuracy (Fig. 2a). Based on considerations of parsimony, we selected lasso (alpha = 1), which was able to classify most individuals correctly (Fig. 2b) and identified four significant proteins (Noggin (NOG), interferon lambda 2 (IFNL2), secreted frizzled-related protein 1 (SFRP1), and midkine (MDK)) based on significance of feature frequency. Two of these proteins (NOG and SFRP1) displayed a significant positive feature coefficient—i.e., increased levels of these proteins were associated with an increased probability of classification as AD (Supplementary Fig. 2a). To confirm these findings, we utilized this signature in a leave-one-out cross-validated logistic regression model in the same ROS cohort, which, as expected, was able to discriminate between ROS ITG AD and CN samples, yielding an AUC = 0.937 (95% CI: 0. 0.846–1.00). The corresponding ROC plot is shown in Fig. 2c. Then, we tested this same signature in an independent cohort, the BLSA, which demonstrated much weaker discriminatory potential, yielding AUC = 0.643 (95% CI: 0.463–0.823) as shown in Fig. 2d.

We additionally tested the ability of the ROS-derived ITG signature to discriminate between AD and CN in BLSA MFG samples. The signature discriminated between AD and CN participants with an AUC of 0.867 (95% CI: 0.746–0.988).

### Brain and blood serum levels of MFG protein signature

To determine differences in levels of SMOC1, NOG, APCS, and NTN1 between AD and CN individuals, we performed proportional odds models separately for each cohort. Comparisons were made for both ROS and BLSA between protein levels in ITG, MFG, and blood serum at the time of sampling concurrent with diagnosis and age-matched controls. Among all 1300 proteins assayed in the MFG, 352 were significantly different (*p* < 0.05) between AD and CN in BLSA participants, and 223 were significantly different between conditions in ROS participants (Supplementary Table 3).

**Fig. 3:**
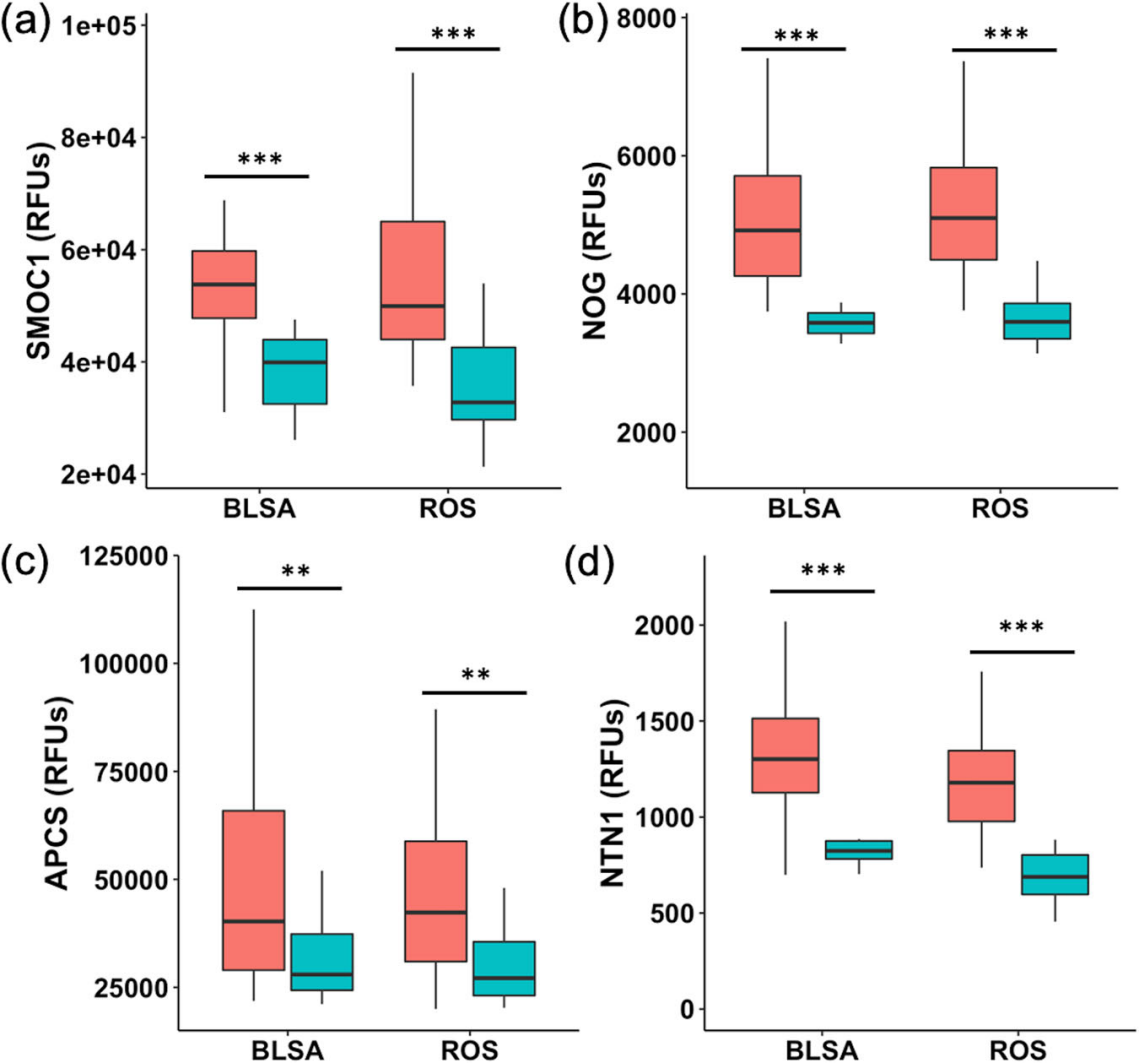
MFG levels of proteins present in the proteomic signature. Comparison of MFG levels of **a** SMOC1, **b** NOG, **c** APCS, and **d** NTN1 between AD (red) and CN (blue) individuals in BLSA and ROS. Protein levels are on the y-axis in relative fluorescence units (RFUs). Statistical significance was calculated using sex and age-adjusted proportional odds models. Horizontal lines within each box represent the mean, and error bars represent the standard deviation. **p < 0.01, ***p < 0.001.

In the MFG brain region in which the proteomic signature was established, all 4 proteins were significantly different between AD and CN individuals in both cohorts at the *p* < 0.01 level (Fig. 3). For all four proteins, levels were higher in AD than in CN individuals in both ROS and BLSA cohorts. In the ITG, the majority of protein comparisons were also significant between AD and CN in both cohorts (Supplementary Fig. 3). Only APCS was not statistically significant (*p* = 0.095) in the ROS ITG, while all other proteins were significantly higher in AD ITG samples compared to CN. In blood serum samples at the time of diagnosis, levels of APCS were higher in AD individuals, and levels of NTN1 were lower compared to CN individuals (Supplementary Fig. 4). These results were significant in the ROS cohort only.

### Brain and blood serum levels of ITG protein signature

We also determined differences in levels of NOG, MDK, SFRP1, and IFNL2 between AD and CN brain samples. In the ITG brain region, in which the signature was established, all four proteins were significantly different in ROS, but only one protein (NOG) was significant in BLSA. SFRP1, IFNL2, and NOG were increased in ROS AD participants, while IFNL2 was reduced. NOG was increased in BLSA AD participants. In blood serum at the time of diagnosis, none of the four proteins were significant in ROS or BLSA participants. Among all 1300 proteins assayed in the ITG, 150 were significantly different between AD and CN participants in the BLSA cohort, and 353 were significantly different in the ROS cohort (Supplementary Table 3).

### Brain and blood serum protein correlations with AD pathology

We also sought to determine if proteins in the MFG signature correlated with Braak and CERAD scores, two measures of AD pathology. We performed partial correlation analyses between ITG and MFG protein levels of each of the four proteins with Braak and CERAD scores separately.

**Fig. 4:**
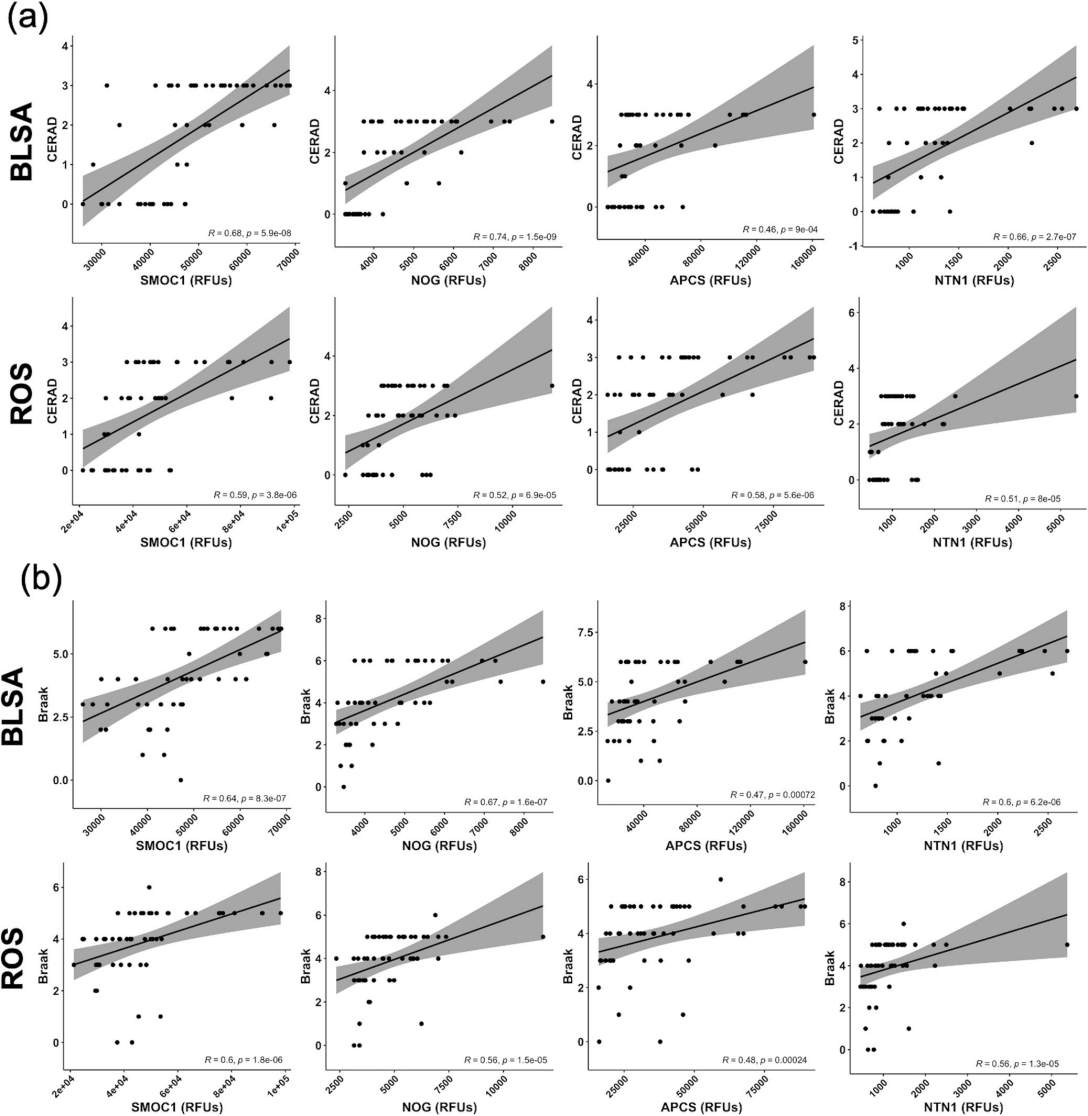
Correlation of MFG protein levels with AD pathology. Partial Spearman correlation, adjusted for sex and age at sampling, between MFG protein levels and **a** CERAD and **b** Braak scores in BLSA and ROS. Protein levels are given in relative fluorescence units (RFUs) on the x-axis. Shading indicates the 95 percent confidence interval of the estimate.

In the MFG, all four proteins included in the signature displayed strong positive correlations with both CERAD and Braak scores (Fig. 4). Similarly, ITG levels of most proteins from the signature displayed strong positive correlations with both scores of AD pathology (Supplementary Fig. 5). The only correlation that did not achieve statistical significance was the association between ITG levels of SMOC1 and Braak (*p* = 0.16).

As in the ITG and MFG, we performed partial correlation analyses between blood serum levels of proteins in the signature at the time of diagnosis with scores of AD pathology. For the most part, correlations between blood serum proteins and AD pathology scores were not significant. However, NOG displayed a positive correlation with CERAD, and NTN1 displayed a negative association with Braak in the BLSA cohort only (Supplementary Fig. 6).

## Discussion

In this study, we utilized a machine learning approach to perform feature reduction with the goal of identifying a parsimonious set of proteins whose expression levels in the brain could accurately discriminate between AD and CN individuals. We utilized two independent cohorts of AD individuals with autopsy brain tissue samples, performing model training and feature selection in ROS and then testing the selected model in BLSA. This methodology allowed us to establish a four-protein signature (SMOC1, APCS, NOG, and NTN1) that accurately discriminated AD and CN MFG brain tissue samples in both ROS and BLSA cohorts. We then compared levels of these four proteins in MFG and ITG brain tissue samples at autopsy, as well as blood serum samples at the time of AD diagnosis. In both brain regions, these proteins consistently differed between AD and CN individuals. Notably, serum concentrations of APCS and NTN1 additionally differed between AD and CN individuals in the ROS cohort at the time of AD diagnosis, suggesting these brain proteomic alterations may extend to the peripheral system. Lastly, we sought to determine the correlation between proteins in this signature and the severity of AD pathology, identifying strong positive correlations between ITG and MFG levels of all four proteins and CERAD and Braak in ROS and BLSA. While relationships between blood serum levels and AD pathology were less consistent, NOG and NTN1 did correlate with AD pathology in BLSA samples.

The use of stringent feature reduction techniques to identify proteins that are highly associated with AD status allows for a more granular exploration of the potential mechanisms of the relationship between proteomic alterations and AD pathogenesis. APCS, or serum amyloid P component, is a pentraxin that has consistently been identified in CSF, as well as bound to amyloid deposits and neurofibrillary tangles, in individuals with AD^12,13^. APCS has been shown to stabilize amyloid plaques, prevent proteolysis, and promotes deposition of amyloid in vitro^14,15^. In animal models and cell culture experiments, APCS has further been shown to induce neuronal apoptosis^16,17^ and plays a role in the immunological response via its effects on macrophage polarization^18^. More systemically, APCS functions alongside CRP as a key mediator of the innate immune response, binding to pattern-recognition sequences on microbes, as well as recognizing damaged tissue^19,20^. Both APCS and CRP activate the complement activation cascade, with APCS binding C1q and C4b proteins^21^. APCS, and other pentraxins, may therefore represent a possible mediator between long-standing inflammation and AD, further contributing to hallmark features of pathology. Indeed, studies have shown that APCS deposition on neurofibrillary tangles occurs prior to the activation of complement, thereby contributing to local inflammation and resultant neuronal loss^22^.

SMOC1 is a member of the SPARC matricellular protein family with a role in the regulation of the interaction between cell matrices via the binding of cell surface receptors^23^. SMOC1 has been discussed as a possible mediator of inflammation, as nitric oxide inhibits SMOC1 function, which in turn results in reduced TGF-β signaling^24^. SMOC1 appears to be consistently identified as a key protein in AD, as multiple proteomics studies have found elevations of SMOC1 in brain tissue and CSF, as well as associations with amyloid pathology^25–27^. Such consistent findings suggest that SMOC1 likely represents a plausible biomarker for AD that is sensitive as a marker of AD pathology. SMOC1 also plays a role as a pro-angiogenic factor^28^, which has frequently been proposed as a biological process perturbed in AD^29^.

NOG is a protein with a role in the development of multiple tissues, including neural cells, and is a member of the TGF-β protein family, acting as an antagonist of bone morphogenetic protein-4 (BMP-4). Prior studies have identified the role of NOG in the regeneration of neurons following injury^30^. Others have found that NOG signaling through BMP changes throughout the lifecourse, finding that age-related increases in BMP signaling are associated with impaired neurogenesis that may be buffered by NOG^31^. Animal studies additionally suggest that NOG can rescue hippocampal neurogenesis and behavioral deficits^32^. However, in this study, we identified an increase in NOG in AD and a positive correlation with pathology, which may suggest that NOG elevation may represent an attempt to promote neurogenesis in response to neuronal loss in AD. Previously, NOG has been found to be hypermethylated in AD, suggesting that genetic modification leading to an imbalance of NOG and BMP-4 may be a feature of disease^33^.

NTN1 belongs to a family of proteins called netrins that play a role in neuronal axon guidance through modulation of microtubular function and additionally function to prevent apoptosis initiation. NTN1 has previously been proposed as a therapeutic for AD after the finding that administration of NTN1 to transgenic mice reduced amyloid beta and improved working memory^34^. Additionally, NTN1 gene expression in blood has previously been found to be reduced in mild cognitive impairment (MCI)^35^. Interestingly, we found NTN1 to be reduced in blood serum in ROS individuals with AD at the time of diagnosis yet increased in BLSA and ROS postmortem samples and positively correlated with AD pathology. This may suggest that early in the disease course, reductions in NTN1 could contribute to AD pathogenesis, while later increases in expression could indicate a response to the disease.

In summary, we have undertaken a unique multivariable regression approach to identify a reduced set of proteins that could discriminate between AD and CN brain tissue samples. We achieved this by deriving a proteomic signature in one cohort, ROS, before testing the discriminatory signature in an independent cohort, BLSA. This signature was identified despite significant demographic differences across cohorts, suggesting that the identified protein signature may be associated with disease independent of the study sample. This process identified four proteins in the MFG brain region that discriminated accurately between AD and CN samples in both cohorts that may represent key features of AD pathogenesis. Despite strong replication in brain tissue, however, the MFG signature failed to replicate in blood serum obtained from participants at a baseline visit. This suggests the signature is unlikely to have significant utility as a serum screening biomarker in pre-clinical populations. It may specifically reflect mechanisms of disease pathophysiology in brain tissue, though exploration of these proteins in cerebrospinal fluid may further evaluate their utility as AD biomarkers.

In contrast, the ITG protein signature identified in ROS failed to discriminate between AD and CN in BLSA. This may be due to a number of demographic differences between the two cohorts, as ROS participants were on average older, more often female, and with shorter disease duration as compared to BLSA. In addition, while CERAD scores, reflective of amyloid pathology, were similar across cohorts, AD participants in the BLSA had, on average, higher Braak scores, reflective of greater accumulated neurofibrillary tangle pathology. The ITG was specifically selected in this study as a brain region vulnerable to tau-related neurofibrillary tangle pathology, while the MFG was selected as a brain region vulnerable to amyloid accumulation^36,37^. Numerous studies have suggested that amyloid beta precedes and may induce tau aggregation, and distinct neurodegenerative alterations occur when tau and amyloid colocalize^38–40^. Our findings of a strong MFG-specific proteomic signature are, therefore, most likely to represent a signature of AD that is closely related to amyloid pathology, while the failure of the ITG signature to replicate may reflect varying severity of neurofibrillary tangle pathology across cohorts. Indeed, SMOC1 and NTN1 were recently identified among the most highly-enriched proteins in amyloid plaques in early-onset AD and individuals with Down syndrome and AD^41^. Similarly, both proteins were also among the most highly-enriched proteins in amyloid plaques in late-onset AD^42^, strongly suggesting the MFG signature is reflective of proteomic alterations related to amyloid plaque formation. In addition, MDK, which was present in the ITG signature that also replicated in the MFG, was identified as a highly-enriched protein in amyloid plaques in both of these prior studies^41,42^.

In sum, we have applied a rigorous statistical methodology to derive an MFG protein signature discriminating between AD and CN participants in two independent cohorts that may have significant specificity to amyloid pathology. This study confirms prior work identifying SMOC1 and NTN1 enrichment within amyloid plaques and extends results to suggest that NOG and APCS are amyloid plaque-related proteins warranting further evaluation. The approach taken here may provide further utility in evaluating larger datasets of blood and CSF samples throughout the course of AD development in order to identify improved biomarkers or earlier targets for therapeutics. Additionally, this approach may be adapted as even greater multi-dimensional datasets emerge, allowing researchers to parse through such datasets for rapid identification of proteins that may most substantially contribute to disease outcomes.

## Methods

### Participants: Baltimore Longitudinal Study of Aging (BLSA)

The National Institute on Aging’s (NIA) BLSA is among the longest-running scientific studies of aging in the United States^43^. This observational study began in 1958 and includes longitudinal, radiological, clinical, and laboratory evaluations of community-dwelling volunteer participants. The individuals included in this study were participants in the autopsy sub-study of the BLSA, which has been described previously^44^. Postmortem brains were inspected by an expert neuropathologist to assess AD pathology. The Consortium to Establish a Registry for Alzheimer’s Disease (CERAD) and Braak criteria were used to assess the severity of AD pathology based on neuritic plaques^45^ and neurofibrillary tangles^46^, respectively, as described previously in ref. ^47^. Clinical diagnosis of dementia and AD have previously been described^48^ and were based on the Diagnostic and Statistical Manual (DSM)-III-R^49^ and the National Institute of Neurological and Communication Disorders and Stroke–Alzheimer’s Disease and Related Disorders Association (NINCDS-ADRDA) criteria, respectively^50^.

Diagnosis and cognitive status were determined at consensus diagnosis conferences using procedures described in detail previously^48^. Briefly, autopsy participants were classified as either AD or CN according to the following criteria: AD participants had a clinical diagnosis of AD or mild cognitive impairment (MCI) within 1 year of death in addition to a postmortem CERAD pathology score >1 (i.e., CERAD B or C); cognitively normal (CN) participants had normal cognition within 1 year of death and a CERAD pathology score ≤1 (i.e., CERAD 0 or A). Demographic characteristics of the BLSA cohort are included in Table 1. The BLSA study protocol has ongoing approval from the Institutional Review Board of the National Institute of Environmental Health Science, the National Institutes of Health, and informed consent was obtained from all participants.

### Participants: Religious Orders Study (ROS)

The Religious Orders Study (ROS) has enrolled Catholic nuns, priests, and brothers from a multitude of communities across the United States since 1994^51^. This longitudinal observational study collects information from clinical, neuroimaging, laboratory, and self-report evaluations of employed and retired community-dwelling individuals. At the time of enrollment, participants are without a diagnosis of known dementia. All participants agreed to organ donation and annual clinical evaluation.

Our sample consisted of a subset of participants from the larger ROS cohort study. All ROS participants provided written informed consent and the study was approved by an Institutional Review Board of Rush University Medical Center. Participants signed an Anatomical Gift Act for organ donation and a repository consent to allow their data and biospecimens to be shared. At each study visit, dementia status was determined by trained clinicians using all cognitive and clinical data blinded to prior years based on NINCDS-ADRDA criteria. A final consensus clinical diagnosis was determined at death, blinded to all neuropathologic data. Autopsies were performed based on standard methods reported using 4% paraformaldehyde-fixed 1-cm sections of brain tissue for neuropathology analyses^52^. Postmortem brains were examined by an expert neuropathologist or trained technician to assess AD pathology. CERAD and Braak criteria were used to assess the severity of AD pathology, as described previously^53^.

Participants were classified into two groups. AD participants had a final clinical diagnosis of AD and an NIA-Reagan score of an intermediate or high likelihood of AD. NIA-Reagan criteria are based on both neuritic plaques (CERAD score) and neurofibrillary tangles (Braak score)^54^. CN participants had a clinical diagnosis of no cognitive impairment (NCI) and an NIA-Reagan score of a low likelihood of AD or no AD. Diagnosis and cognitive status were determined based on a three-stage process described previously^51^, which included a medical history, neurological examination, neuropsychiatric testing, and review of neuroimaging data when present. At death, clinical data were reviewed in order to make a likely clinical diagnosis.

### Brain tissue collection and homogenization

BLSA (AD = 31, CN = 19) and ROS (AD = 31, CN = 22) brain tissue samples were selected from two a priori specified regions: the inferior temporal gyrus (ITG) and middle frontal gyrus (MFG). The ITG was selected to represent a brain region vulnerable to neurofibrillary tangle deposition and the MFG was selected to represent a region vulnerable to β-amyloid accumulation^36,37^. Approximately 4-mm-diameter tissue punches were extracted from the cortical surface of the brain tissue regions using a sterile technique and stored at −80 °C prior to proteomic assays.

To ~10 mg of brain tissue, 110 µl of T-PER (tissue protein extraction reagent) (Thermo Fisher Scientific, USA) with 2 µl of Halt™ Protease and Phosphatase Inhibitor cocktail (Thermo Fisher Scientific, USA) was added and placed in a CKMix grinding tube, containing soft tissue homogenizing lysis beads (a mix of 1.4 mm and 2.8 mm ceramic (zirconium oxide) beads) (Bertin Technologies, San Quentin, France). The tubes were placed in the Precellys Evolution tissue homogenizer (Bertin Technologies, San Quentin, France) and homogenized for two 30 s cycles of 6500 rpm and a 30 s rest in between. The homogenate was removed and placed in an Eppendorf tube and centrifuged at 16,000×*g* for 5 min at 4 °C. The supernatant was removed and centrifuged a second time for 10 min at 16,000×*g* at 4 °C. The supernatant was collected at 4 °C, 2.5 µl was aliquoted, and protein quantitation was carried out using a MicroBCA Protein Assay Kit (Thermo Scientific, USA). The total protein concentration was determined, and the samples were diluted to a final volume of 200 µg/ml with PBS 1X and stored at −80 °C until analysis.

### Blood serum collection

Blood serum samples were collected from BLSA participants (AD = 26, CN = 20) at time of AD diagnosis, 43 of whom were included in the autopsy sample, at the NIA Clinical Research Unit in Harbor Hospital, Baltimore. Details on collection and processing have been published previously^55^. Briefly, venous blood samples were collected between 6 and 7 a.m. following an overnight fast. Serum samples were aliquoted into 0.5-mL volume in Nunc cryogenic tubes and stored at −80 °C until further use. Samples were not subject to any freeze–thaw cycles prior to proteomic assays. Additional details on sample selection have been published previously^55^. ROS participants (AD = 29, CN = 22) provided blood serum at the time of AD diagnosis, all of whom were included in the autopsy sample, as described previously^56^.

### SOMAscan proteomic quantification

Sample total protein was adjusted to 16 μg/mL in SB17T buffer (40 mM HEPES, 125 mM NaCl, 5 mM KCl, 5 mM MgCl_2_, 1 mM EDTA, 0.05% Tween-20 at pH 7.5). Proteomic profiles for 1322 SOMAmers were assessed using the 1.3 K SOMAscan assay (SomaLogic, Inc., Boulder, CO, USA) at the Trans‐NIH Center for Human Immunology and Autoimmunity, and Inflammation (CHI), National Institute of Allergy and Infectious Disease, National Institutes of Health (Bethesda, MD, USA). The SOMAscan assay platform includes 1322 SOMAmer Reagents, of which 12 are hybridization controls, five are viral proteins, and five are non-specifically-targeted SOMAmers. As a result, analyses spanned 1300 SOMAmer Reagents. The proteins targeted by SOMAmers included in this assay are shown in Supplementary Table 1. The experimental procedure for proteomic assessment and normalization has been previously reported^57^. Briefly, the SOMAscan assay uses SOMAmers to translate protein concentrations into measurable DNA signals, which can be quantified using standard DNA detection procedures. This is achieved by affinity binding and biotin capture on streptavidin beads. The DNA concentrations obtained from this method are reported as relative fluorescence units (RFUs), resulting from fluorescent SOMAmer hybridized to its complementary probe on an Agilent array, and are directly proportional to the reported relative abundance of SOMAmer Reagents. The data normalization process includes hybridization, control normalization, median signal normalization, and calibration normalization, as previously described in refs. ^57,58^.

Study/cohort-specific samples were run in the same batch. Within the study/cohort, sample ordering was randomized by disease (AD and CN), for brain tissue by brain region (ITG and MFG), sex, and age. SomaScan reports protein abundances measured in RFU with no missing or below limit of detection (LOD) values. Following standard SomaScan normalization procedures^58^, RFU data were processed using hybridization normalization, median signal normalization, plate-scale normalization, and inter-plate calibration, which were designed to remove nuisance variability due to differences in loading volume, leaks, washing conditions, microarray hybridization, and other sources of intra- and inter-plate technical effects. Additional quality checks based on principal component analysis were performed at each normalization step; the fully normalized data used for downstream analysis showed no evidence of significant technical bias. Outliers were not excluded.

### Statistical analysis: demographic characteristics

Demographic characteristics are summarized in Table 1. Comparisons between the BLSA and ROS cohorts were performed using two-sample *t*-tests for continuous variables and chi-square tests for categorical variables.

### Statistical analysis: brain proteomic signature identification

To establish a brain proteomic signature discriminating AD and CN brain samples, we utilized a two-step process in which we first identified a signature in ROS and then validated the signature independently in BLSA. We performed the signature identification step separately in ROS ITG and MFG brain tissue samples utilizing binomial classification in eNetXplorer^8^, an R package, which tests the accuracy and significance of a family of elastic net generalized linear models ranging from ridge regression (alpha = 0) to lasso regression (alpha = 1)^7^. For analysis in eNetXplorer, the outcome was a binary group variable (AD vs. CN) with predictors of log_10_-transformed aptamer relative fluorescence units (RFU) in the brain and covariates of sex and age at death. The elastic net mixing parameter alpha was scanned from 0 to 1 in 0.2 intervals. The alpha model best discriminating between AD and CN in ROS samples was selected based on overall performance assessed as a function of the fivefold cross-validated quality function (accuracy) and the empirical *p* value estimated from comparing the model against a statistical ensemble of null models generated by random permutations of the response (i.e., AD and CN class labels). This procedure required the evaluation of a large number of elastic net realizations arising from 500 randomly generated fivefold cross-validation runs, each of them compared against 250 null model permutations, in which participant class labels (AD or CN) are randomly assigned. Model performance is quantified by a quality function (i.e., classification accuracy), and the comparison between the model performance and null model performance distribution is quantified with an empiric *p* value. To identify proteins defining a discriminative proteomic signature, we selected proteins with a significant (*p* value <0.05) feature frequency (frequency with which a protein was selected by the model) or feature coefficient in the selected alpha model. Proteins identified in this manner by eNetXplorer are those that discriminate most accurately between AD and CN brain samples in ROS. False-discovery rate corrections were not applied to selected protein features as such methodologies assume statistical independence, whereas eNetXplorer preserves the data covariance structure across feature space.

After identifying proteins accurately discriminating between AD and CN brain samples in ROS with eNetXplorer, we validated the discriminative ability of these proteins by applying leave-one-out cross-validation (LOOCV) with logistic regression models in an independent cohort of BLSA brain samples. In LOOCV, one sample is held out from the data as a test set and all other samples are used in the training set. This procedure is repeated until all samples in the dataset have been used as the test set^59^. LOOCV was performed separately within each brain region: i.e., the ROS ITG brain proteomic signature was applied to BLSA ITG tissue samples, and the ROS MFG signature was applied to BLSA MFG samples. Prior to model evaluation, we performed three interquartile range (IQR) winsorization, in which protein values outside the 3 IQR range were “winsorized” and set equal to the three IQR limit^60^. Logistic regression models included the group outcome (AD vs. CN), and predictors included levels of proteins in the brain proteomic signature, as well as covariates—sex and age. Results of LOOCV were visualized as receiver operating characteristic (ROC) curves, from which we calculated area under the curve (AUC) with 95 percent confidence intervals.

### Statistical analysis: protein abundance comparisons

To identify differences in levels of proteins identified by eNetXplorer between AD and CN participants, we used proportional odds models^61^, a generalization of the non-parametric Wilcoxon and Kruskal-Wallis tests. All proportional odds models included ranked protein levels (outcome), the group predictor (AD vs. CN), and covariates—the age at sampling and sex. Models were run separately for ROS and BLSA cohorts. For each cohort, three sets of cross-sectional proportional odds models were performed, one for each protein sampling matrix: ITG brain tissue, MFG brain tissue, and blood serum at the time of diagnosis. The significance for between-group comparisons was set as *p* < 0.05.

### Statistical analysis: associations with AD pathology

We also sought to determine if blood serum and brain levels of proteins in the brain proteomic signature were associated with the severity of AD pathology in the ITG and MFG. As in prior work^62^, we examined partial Spearman correlations of CERAD and Braak scores with ranked aptamer values, controlling for covariates –sex and age at sampling. Correlations were performed separately in ROS and BLSA for ITG, MFG, and serum protein levels at the time of samples concurrent (<1 year) with AD diagnosis and age-matched controls. A significant (at a *p* value threshold of 0.05) positive correlation indicated that a higher concentration of the protein was associated with higher AD pathology (i.e., higher CERAD or Braak scores) and, conversely, a significant negative correlation indicated that a lower concentration of the protein was associated with higher AD pathology.

### Reporting summary

Further information on research design is available in the Nature Research Reporting Summary linked to this article.

## Supplementary information

Supplementary Material

Reporting Summary

Supplementary Table 1 and 3

## Data Availability

NIH and BLSA policy prohibits the deposition of the proteomic data associated with this manuscript in a public repository. However, unprocessed SomaLogic proteomic data from the Baltimore Longitudinal Study of Aging (BLSA) are available to researchers and can be requested at https://blsa.nih.gov/researchers, in accordance with BLSA and NIH policy. SomaLogic proteomic data from the Religious Orders Study (ROS) are available to researchers at www.radc.rush.edu.

## Code availability

All analyses were performed in R Studio version 1.3.1073 using publicly-available packages as described in the Methods. Requests for code utilized in this manuscript may be made to the corresponding author.
