# Supplementary material for: Unbiased proteomics and multivariable regularized regression techniques identify SMOC1, NOG, APCS, and NTN1 in an Alzheimer’s disease brain proteomic signature
Source: NPJ Aging. 2023 Jul 6;9(1):18. doi: 10.1038/s41514-023-00112-6 (PMC10326005; doi:10.1038/s41514-023-00112-6)
Supplement: Supplementary file 1 — Supplementary Material [file 41514_2023_112_MOESM1_ESM.pdf]

Supplementary Material

Supplementary Table 2. Demographic characteristics, individuals with blood serum samples.

Baltimore Longitudinal Study of Aging (BLSA)			
	Total Sample N = 46	AD N = 26	CN N = 20
Age at diagnosis, mean (SD)	72.85 (11.01)†	72.78 (10.73)†	72.94 (11.66)†
Sex, n (% female)	18 (39.13)†	12 (46.15)†	6 (30.00)†
Race, n (% white)	43 (93.48)	26 (100)*	17 (85.00)*
CERAD, mean (SD)	1.65 (1.35)	2.77 (0.43)*	0.20 (0.41)*
Braak, mean (SD)	4.14 (1.69)	5.12 (1.14)*	2.55 (1.10)*

Religious Orders Study (ROS)			
	Total Sample N = 51	AD N = 29	CN N = 22
Age at diagnosis, mean (SD)	81.21 (5.94)†	82.13 (5.56)†	80.00 (6.33)†
Sex, n (% female)	36 (70.59)†	23 (79.31)*†	13 (59.09)*†
Race, n (% white)	51 (100.00)	29 (100.00)	22 (100.00)
CERAD, mean (SD)	1.35 (1.25)	2.14 (0.99)*	0.32 (0.65)*
Braak, mean (SD)	3.61 (1.31)	4.14 (1.22)*	2.82 (1.30)*

* $p < 0.05$ comparing AD and CN within cohort

† $p < 0.05$ comparing BLSA to ROS (i.e., AD in BLSA compared to AD in ROS)

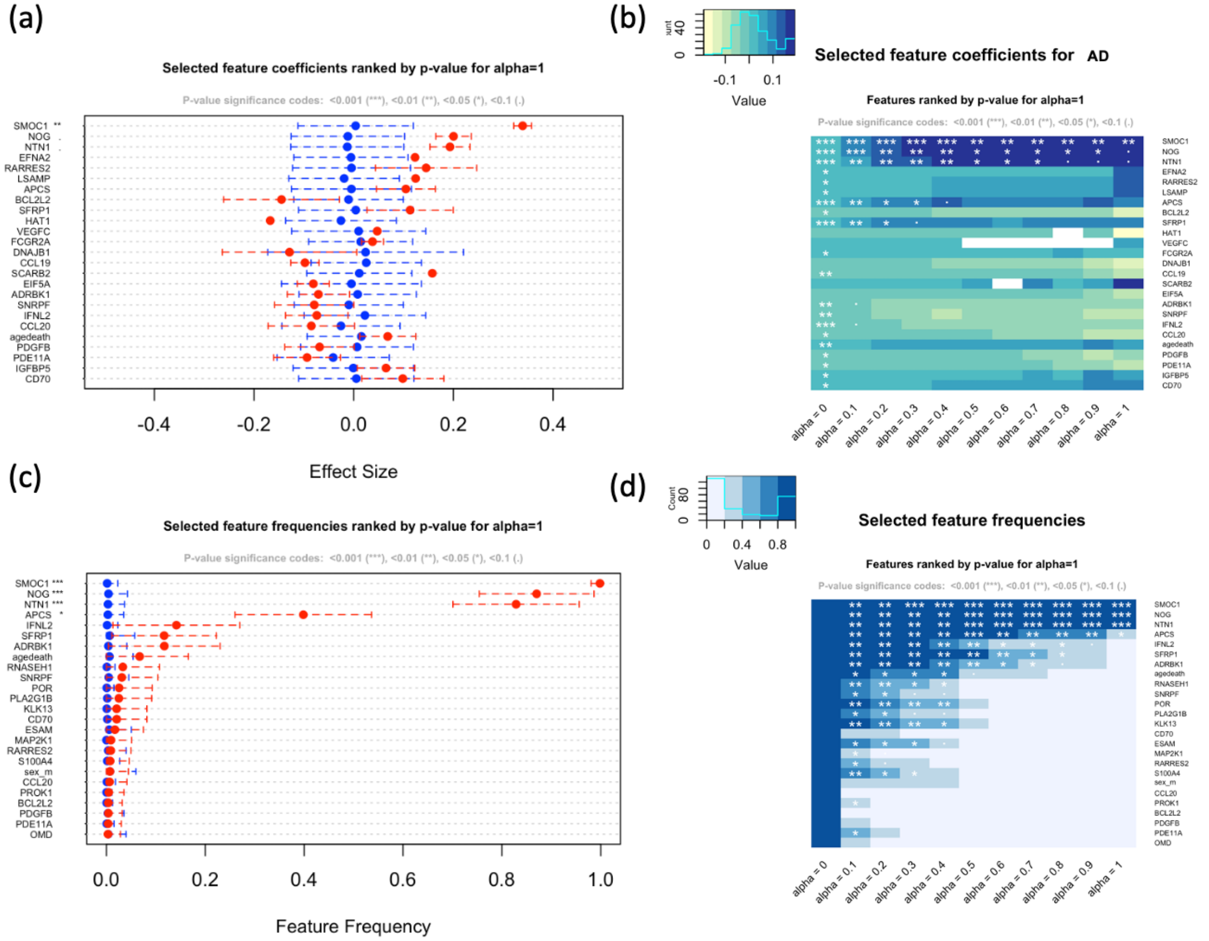

Supplementary Figure 1. Feature coefficients and frequencies for ROS-derived MFG signature. (a) Caterpillar plot indicating the effect size (feature coefficient) of each protein contributing to predictive models in eNetXplorer. Blue dots indicate the average effect size derived from 250 randomly-permutated null models, with blue dashes indicating the 95 percent confidence interval for effect size in null models. Red lines indicate the average effect size from 500 cross-validated models at the *alpha* = 1 level, with red dashes indicating the 95 percent confidence interval for effect size in the model. (b) Heatmap indicating the value and significance of feature coefficients for the 25 top-ranked proteins across *alpha* values. (c-d) Analogously to panels (a-b), top-ranked proteins are here shown based on feature frequency as selection criterion.

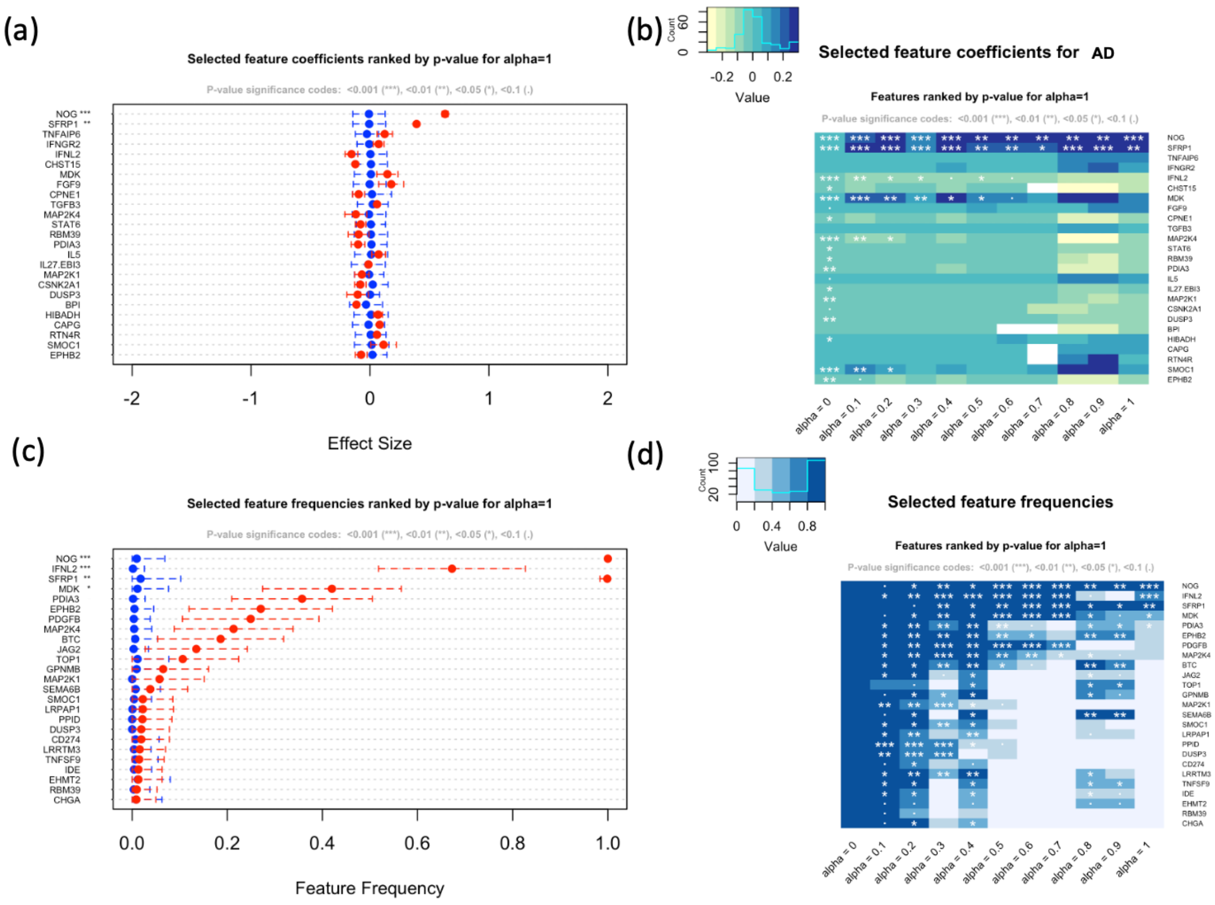

Supplementary Figure 2. Feature coefficients and frequencies for ROS-derived ITG signature. (a) Caterpillar plot indicating the effect size (feature coefficient) of each protein contributing to predictive models in eNetXplorer. Blue dots indicate the average effect size derived from 250 randomly-permutated null models, with blue dashes indicating the 95 percent confidence interval for effect size in null models. Red lines indicate the average effect size from 500 cross-validated models at the *alpha* = 1 level, with red dashes indicating the 95 percent confidence interval for effect size in the model. (b) Heatmap indicating the value and significance of feature coefficients for the 25 top-ranked proteins across *alpha* values. (c-d) Analogously to panels (a-b), top-ranked proteins are here shown based on feature frequency as selection criterion.

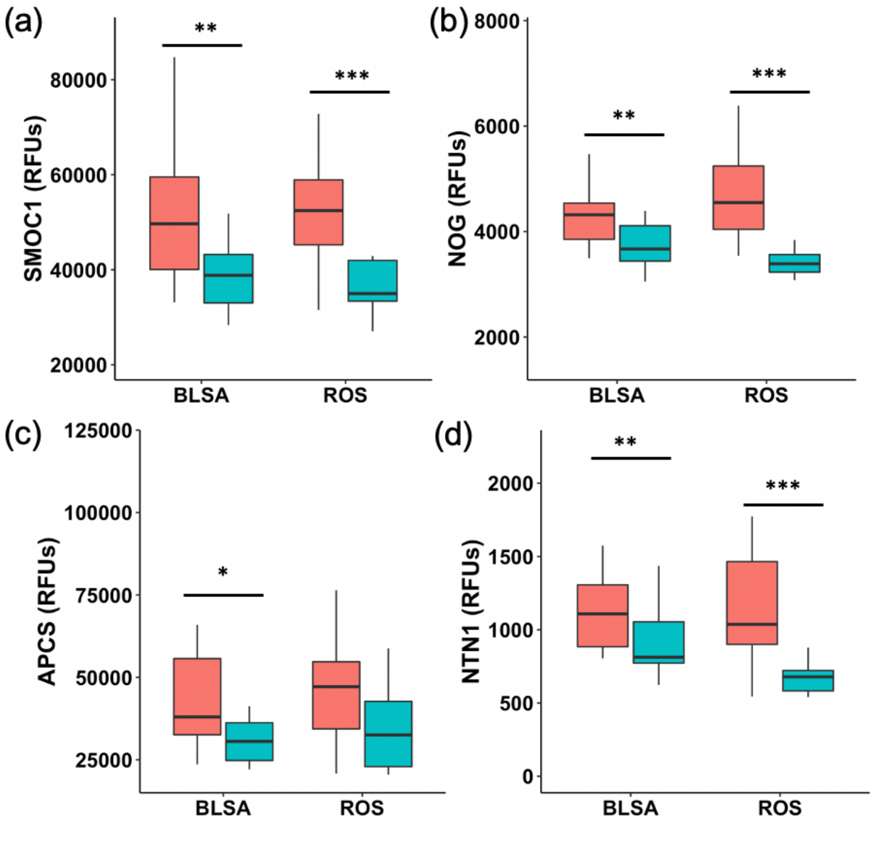

Supplementary Figure 3. ITG protein levels of proteomic signature. ITG levels of (a) SMOC1, (b) NOG, (c) APCS, and (d) NTN1 between AD (red) and CN (blue) individuals in BLSA and ROS. Protein levels are on the y-axis in relative fluorescence units (RFUs). Statistical significance was calculated using sex and age-adjusted proportional odds models. Center lines indicate the mean, and error bars indicate the standard deviation. * $p < 0.05$, ** $p < 0.01$, *** $p < 0.001$

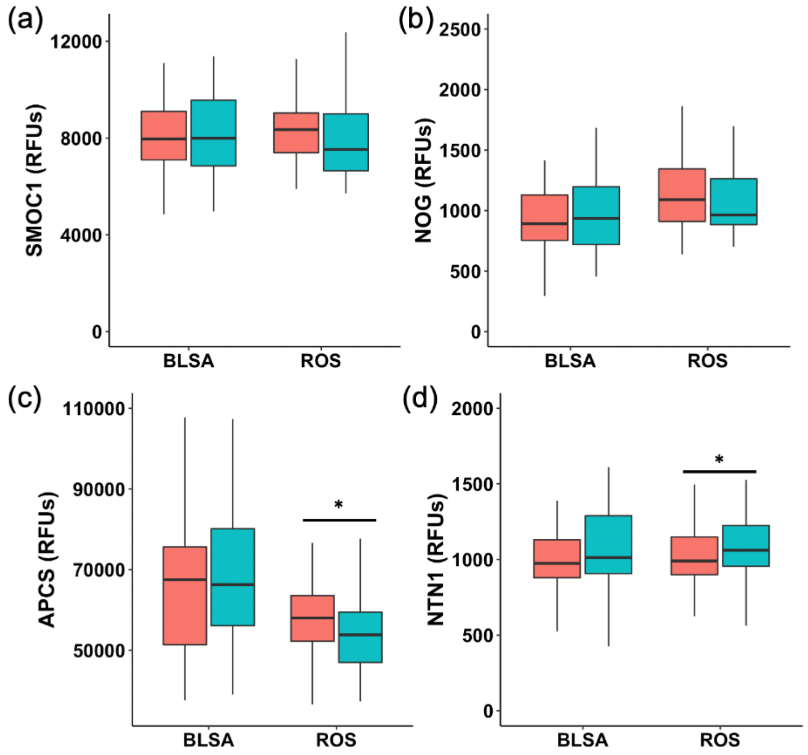

Supplementary Figure 4. Blood serum protein levels of proteomic signature at time of AD diagnosis. Blood serum levels of (a) SMOC1, (b) NOG, (c) APCS, and (d) NTN1 between AD (red) and CN (blue) individuals in BLSA and ROS. Protein levels are on the y-axis in relative fluorescence units (RFUs). Statistical significance was calculated using sex and age-adjusted proportional odds models. Center lines indicate the mean, and error bars indicate the standard deviation. * $p < 0.05$, ** $p < 0.01$, *** $p < 0.001$

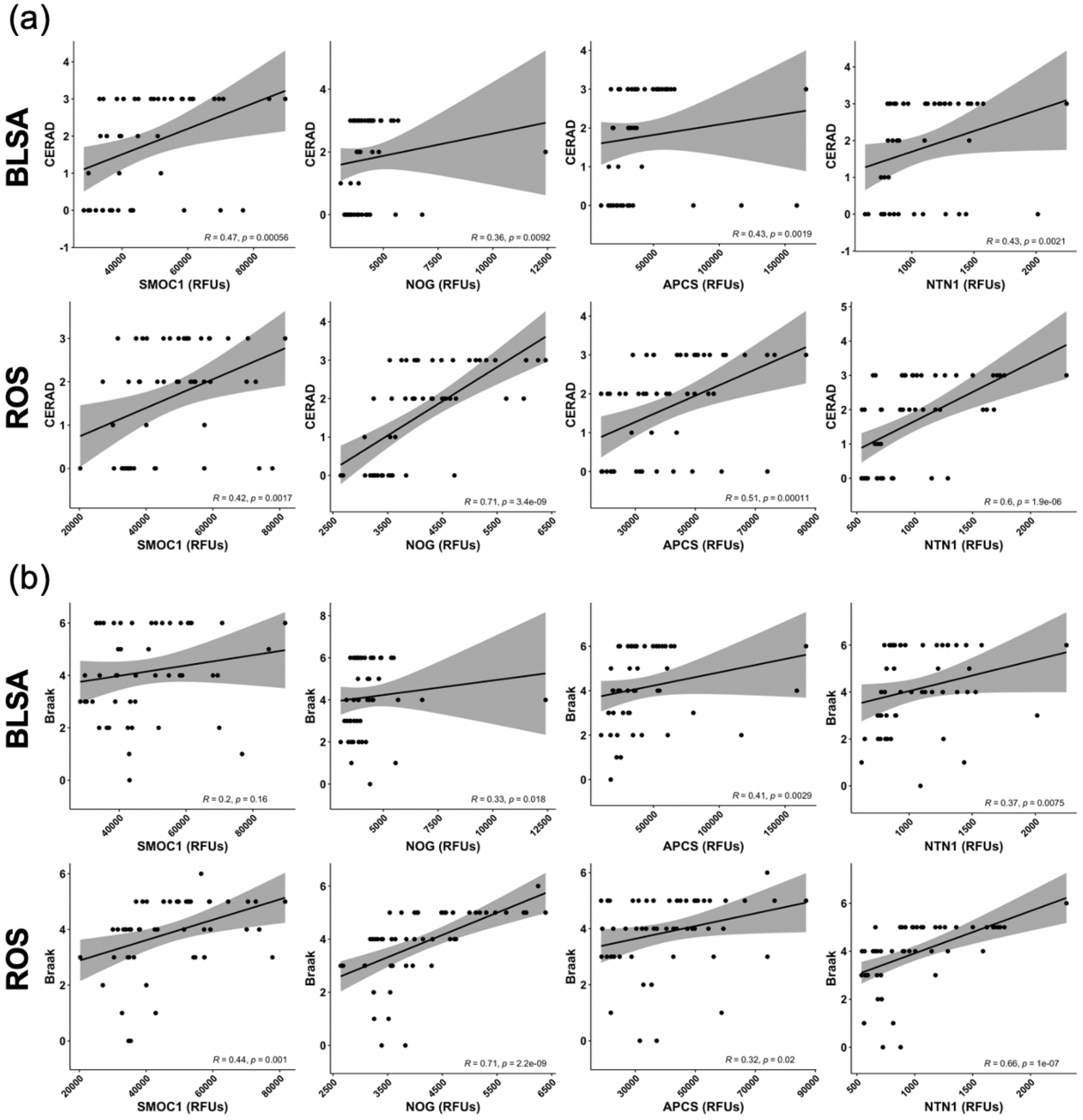

Supplementary Figure 5. Correlation of ITG protein levels with AD pathology. Partial Spearman correlation, adjusted for sex and age at sampling, between ITG protein levels and (a) CERAD and (b) Braak scores in BLSA and ROS. Protein levels are given in relative fluorescence units (RFUs) on the x-axis. Shading indicates the 95 percent confidence interval of the estimate.

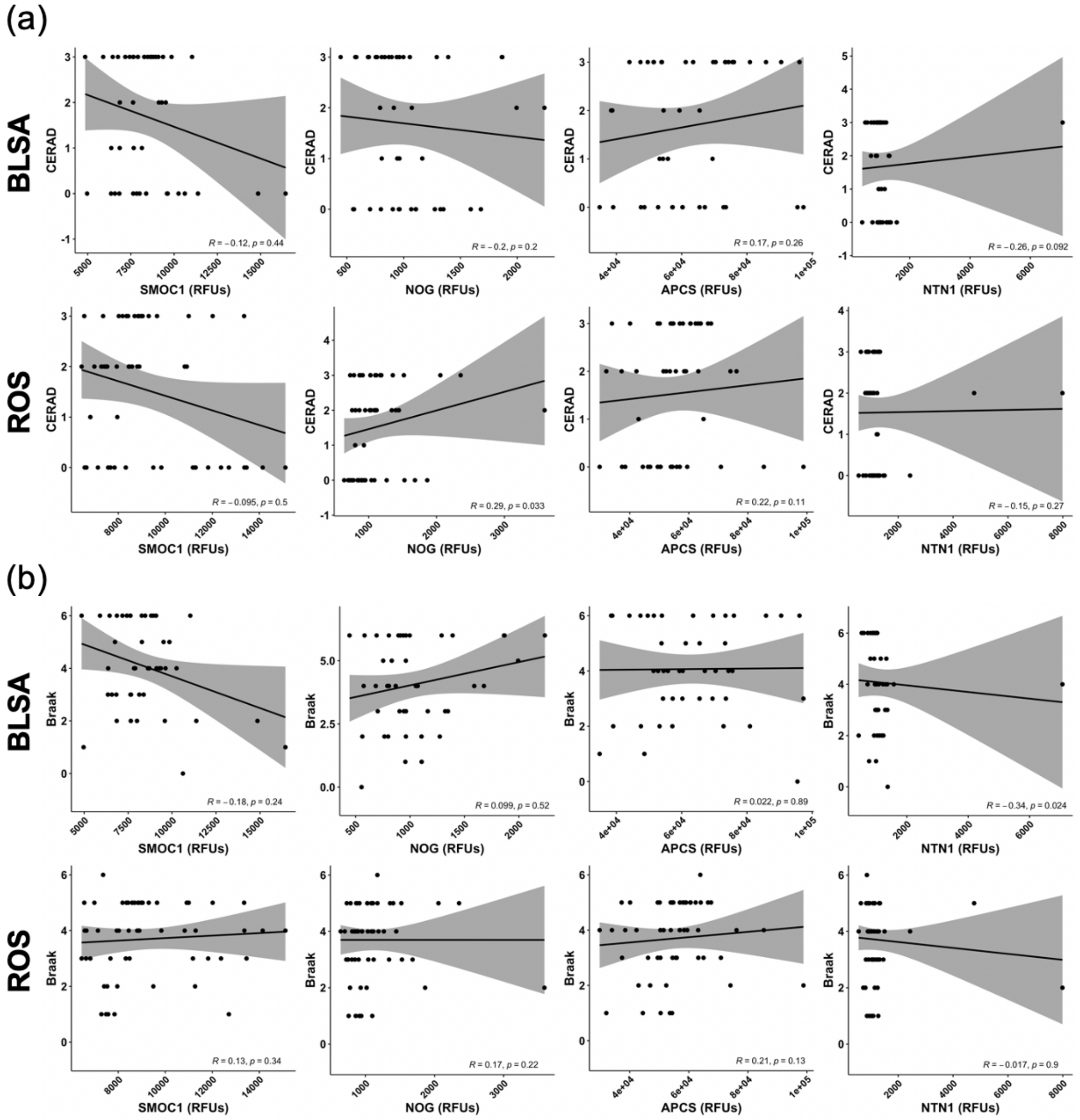

Supplementary Figure 6. Correlation of blood serum protein levels at time of diagnosis with AD pathology. Partial Spearman correlation, adjusted for sex and age at sampling, between blood serum protein levels and (a) CERAD and (b) Braak scores in BLSA and ROS. Protein levels are given in relative fluorescence units (RFUs) on the x-axis. Shading indicates the 95 percent confidence interval of the estimate.
