# Supplementary material for: Unbiased proteomics and multivariable regularized regression techniques identify SMOC1, NOG, APCS, and NTN1 in an Alzheimer’s disease brain proteomic signature
Source: NPJ Aging. 2023 Jul 6;9(1):18. doi: 10.1038/s41514-023-00112-6 (PMC10326005; doi:10.1038/s41514-023-00112-6)
Supplement: Supplementary file 2 — Reporting Summary [file 41514_2023_112_MOESM2_ESM.pdf]

nature portfolio

Corresponding author(s): Madhav Thambisetty
thambisettym@mail.nih.gov
Jackson A. Roberts
jar2344@cumc.columbia.edu

Last updated by author(s): May 10, 2023

Reporting Summary

Nature Portfolio wishes to improve the reproducibility of the work that we publish. This form provides structure for consistency and transparency in reporting. For further information on Nature Portfolio policies, see our Editorial Policies and the Editorial Policy Checklist.

Statistics

For all statistical analyses, confirm that the following items are present in the figure legend, table legend, main text, or Methods section.

n/a	Confirmed	
☐	☒	The exact sample size (*n*) for each experimental group/condition, given as a discrete number and unit of measurement
☐	☒	A statement on whether measurements were taken from distinct samples or whether the same sample was measured repeatedly
☐	☒	The statistical test(s) used AND whether they are one- or two-sided<br>*Only common tests should be described solely by name; describe more complex techniques in the Methods section.*
☐	☒	A description of all covariates tested
☐	☒	A description of any assumptions or corrections, such as tests of normality and adjustment for multiple comparisons
☐	☒	A full description of the statistical parameters including central tendency (e.g. means) or other basic estimates (e.g. regression coefficient) AND variation (e.g. standard deviation) or associated estimates of uncertainty (e.g. confidence intervals)
☐	☒	For null hypothesis testing, the test statistic (e.g. *F*, *t*, *r*) with confidence intervals, effect sizes, degrees of freedom and *P* value noted<br>*Give P values as exact values whenever suitable.*
☒	☐	For Bayesian analysis, information on the choice of priors and Markov chain Monte Carlo settings
☐	☒	For hierarchical and complex designs, identification of the appropriate level for tests and full reporting of outcomes
☐	☒	Estimates of effect sizes (e.g. Cohen's *d*, Pearson's *r*), indicating how they were calculated

Our web collection on statistics for biologists contains articles on many of the points above.

Software and code

Policy information about availability of computer code

Data collection	No software was used.
Data analysis	R Studio version 1.3.1073 was used for all analyses with publicly-available analysis packages.

For manuscripts utilizing custom algorithms or software that are central to the research but not yet described in published literature, software must be made available to editors and reviewers. We strongly encourage code deposition in a community repository (e.g. GitHub). See the Nature Portfolio guidelines for submitting code & software for further information.

Data

Policy information about availability of data

All manuscripts must include a data availability statement. This statement should provide the following information, where applicable:

- Accession codes, unique identifiers, or web links for publicly available datasets
- A description of any restrictions on data availability
- For clinical datasets or third party data, please ensure that the statement adheres to our policy

NIH and BLSA policy prohibits deposition of the proteomic data associated with this manuscript in a public repository. However, unprocessed SomaLogic proteomic data from the Baltimore Longitudinal Study of Aging (BLSA) are available to researchers and can be requested at https://blsa.nih.gov/researchers, in accordance with BLSA and NIH policy. SomaLogic proteomic data from the Religious Orders Study (ROS) are available to researchers at www.radc.rush.edu.

Research involving human participants, their data, or biological material

Policy information about studies with human participants or human data. See also policy information about sex, gender (identity/presentation), and sexual orientation and race, ethnicity and racism.

Reporting on sex and gender	Biological sex was utilized and reported for all participants.
Reporting on race, ethnicity, or other socially relevant groupings	Race was reported for all participants in this study. Race was not used as a covariate in analyses as this was a fairly homogeneous cohort with low numbers of non-white individuals. Other socioeconomic data was not included in this analysis.
Population characteristics	The BLSA cohort was on average 86.4 years old at time of death, 42% female, and 96% white. The ROS cohort was on average 90.6 years old at time of death, 75% female, and 100% white.
Recruitment	The Religious Orders Study (ROS) has enrolled Catholic nuns, priests, and brothers from a multitude of communities across the U.S. since 1994. This longitudinal observational study collects information from clinical, neuroimaging, laboratory, and self-report evaluations of employed and retired community-dwelling individuals. At the time of enrollment, participants are without a diagnosis of known dementia. The National Institute on Aging's (NIA) BLSA is among the longest running scientific studies of aging in the U.S. This observational study began in 1958 and includes longitudinal, radiological, clinical, and laboratory evaluations of community-dwelling volunteer participants. The individuals included in this study were participants in the autopsy sub-study of the BLSA, which has been described previously
Ethics oversight	All ROS participants provided written informed consent and the study was approved by an Institutional Review Board of Rush University Medical Center. Participants signed an Anatomical Gift Act for organ donation and a repository consent to allow their data and biospecimens to be shared. The BLSA study protocol has ongoing approval from the Institutional Review Board of the National Institute of Environmental Health Science, National Institutes of Health, and informed consent was obtained from all participants.

Note that full information on the approval of the study protocol must also be provided in the manuscript.

Field-specific reporting

Please select the one below that is the best fit for your research. If you are not sure, read the appropriate sections before making your selection.

☒ Life sciences ☐ Behavioural & social sciences ☐ Ecological, evolutionary & environmental sciences

For a reference copy of the document with all sections, see nature.com/documents/nr-reporting-summary-flat.pdf

Life sciences study design

All studies must disclose on these points even when the disclosure is negative.

Sample size	Sample sizes were predetermined as the subset of participants in each cohort with SomaLogic proteomic data in brain tissue samples.
Data exclusions	No data were excluded from the analyses.
Replication	In order to aid in replication, we undertook analyses in 2 distinct cohorts, using the ROS cohort as a test set and the BLSA condition as a validation set. This allowed us to derive our protein signature in one cohort and validate in a second independent cohort of participants, thus aiding external validity and capacity for replication.
Randomization	Randomization was not performed for this cohort study.
Blinding	During data collection and processing, researchers were blinded to participant condition. During unsupervised machine learning analysis, researchers were not blinded to participant classification.

Reporting for specific materials, systems and methods

We require information from authors about some types of materials, experimental systems and methods used in many studies. Here, indicate whether each material, system or method listed is relevant to your study. If you are not sure if a list item applies to your research, read the appropriate section before selecting a response.

Materials & experimental systems

n/a	Involved in the study	
☒	☐	Antibodies
☒	☐	Eukaryotic cell lines
☒	☐	Palaeontology and archaeology
☒	☐	Animals and other organisms
☒	☐	Clinical data
☒	☐	Dual use research of concern
☒	☐	Plants

Methods

n/a	Involved in the study	
☒	☐	ChIP-seq
☒	☐	Flow cytometry
☒	☐	MRI-based neuroimaging
